# Safety and Effectiveness of Ultrasound-Guided Carpal Tunnel Release: A Systematic Review

**DOI:** 10.3390/jcm15176675

**Published:** 2026-08-28

**Authors:** Marcin Piwnik, Klaudia Marek, Adam Kwapisz, Elżbieta Miller

**Affiliations:** 1 Department of Neurological Rehabilitation, Medical University of Lodz, Milionowa 14, 93-113 Lodz, Poland; elzbieta.dorota.miller@umed.lodz.pl; 2 Clinic of Orthopedics and Pediatric Orthopedics, Medical University of Lodz, 92-213 Lodz, Poland; adam.kwapisz@umed.lodz.pl

**Keywords:** carpal tunnel syndrome, minimally invasive surgical procedures, ultrasonography

## Abstract

**Background**: Carpal tunnel syndrome is the most common entrapment neuropathy of the upper limb and an important cause of pain, sensory disturbance, and hand dysfunction. Open and endoscopic carpal tunnel release are effective, but they may be associated with scar-related pain, wound complications, and delayed return to work. Ultrasound-guided techniques have been developed to reduce soft-tissue trauma, enable real-time visualization of neurovascular structures, and facilitate faster recovery. **Methods**: A systematic search of PubMed, PubMed Central, and Scopus was conducted according to PRISMA guidelines for English-language studies published over the last 14 years. Eligible studies reported ultrasound-guided carpal tunnel release techniques and/or compared them with open, mini-open, or endoscopic procedures. Data on procedural technique, study population, follow-up, clinical outcomes, complications, and methodological characteristics were extracted and qualitatively synthesized. **Results**: Twenty-eight studies were included. Sample sizes ranged from 14 to 152 patients, and follow-up ranged from 3 to 74 months. The most frequently reported ultrasound-guided approaches were device-assisted carpal tunnel release, thread carpal tunnel release, and percutaneous carpal tunnel release. Most studies reported clinically meaningful improvement in symptoms and hand function, small incisions, rapid return to daily activities, and low rates of serious complications. Reported adverse events were mostly minor and transient. However, the evidence was limited by small samples, heterogeneous outcomes, single-center designs, limited long-term follow-up, and potential conflicts of interest. **Conclusions**: Ultrasound-guided carpal tunnel release appears to be a promising, minimally invasive alternative to traditional surgery, though current evidence is of low certainty. Larger independent comparative trials with standardized outcomes and long-term follow-up are needed.

## 1. Introduction

Carpal tunnel syndrome (CTS) is the most common entrapment neuropathy of the upper extremity, affecting approximately 2–4% of the general population [1,2,3]. It causes pain, paresthesia, nocturnal symptoms, and progressive hand dysfunction; in advanced cases, thenar weakness and atrophy may occur. Although conservative treatment is appropriate in mild-to-moderate disease, many patients with persistent or severe symptoms ultimately require surgical decompression [4,5,6]. This broader view of median nerve entrapment—in which extra-tunnel factors and impaired nerve mobility contribute to symptomatology—provides relevant anatomical context for procedures performed under ultrasound guidance, where the nerve and its surrounding fascial planes are visualized dynamically rather than assessed by static compression alone.

Traditional open carpal tunnel release and endoscopic carpal tunnel release are both mature and effective procedures [7,8,9,10,11,12,13]. However, their broader use is limited by practical and clinical constraints: relatively high procedural and perioperative costs, long waiting times for surgery in many healthcare systems, and postoperative morbidity (e.g., scar tenderness, pillar pain, and wound problems), which may delay functional recovery and return to work. As CTS prevalence and surgical demand increase, these limitations become more relevant for both patients and providers.

These challenges support the search for less invasive, resource-efficient alternatives. Ultrasound-guided carpal tunnel release has emerged as a promising option because it enables real-time visualization of critical neurovascular structures and percutaneous division of the transverse carpal ligament through very small incisions, potentially reducing soft-tissue trauma and recovery time [14,15,16]. Carpal tunnel release with ultrasound guidance (CTR-US) is the umbrella term for any percutaneous or micro-invasive division of the transverse carpal ligament (TCL) performed under continuous real-time sonographic visualization of the median nerve and its recurrent motor and palmar cutaneous branches, the ulnar neurovascular bundle, and the superficial palmar arch, rather than under direct open or endoscopic vision. Thread carpal tunnel release (TCTR), introduced by Guo et al., divides the TCL without a scalpel by percutaneously looping a cutting thread around the ligament through two needle punctures (wrist entry and palmar exit) and drawing the ends back and forth in Gigli-saw fashion, so that transection occurs only within the thread loop, and adjacent structures are spared [17,18]. Ultra-minimally invasive ultrasound-guided release (UMIU-CTR), described by Rojo-Manaute and Capa-Grasa, employs a 1 mm access port through which a hook knife divides the TCL retrogradely while preserving the fascial layer immediately palmar to the ligament—the feature from which the “ultra-minimally invasive” designation derives [13,14]. The SX-One MicroKnife (Sonex Health), subsequently marketed as UltraGuideCTR, is a single-use handheld instrument introduced through a <1 cm wrist incision in which two inflatable balloons displace the median nerve and flexor tendons away from a centrally housed retractable hook blade before retrograde transection of the TCL, after which the same device serves as a probe to confirm completeness of release [19,20,21].

Several systematic reviews of ultrasound-guided release have been published, but each covers a restricted segment of the field: percutaneous release only, with a search closing in 2021 [22]; ultrasound-guided injection and release analyzed together [23]; three Spanish randomized trials of a single hook-knife technique [24]; and, most recently, a pooled analysis of 43 studies limited to complete transverse carpal ligament release in which CTR-US was treated as one intervention [25]. None reports outcomes separately for the individual techniques now in clinical use—UMIU-CTR, thread release, percutaneous release, and device-assisted systems—although these differ substantially in instrumentation and training requirements, and none systematically appraises the industry involvement underlying much of the evidence. The present review addresses both gaps.

Based on this, the objective of this systematic review is to evaluate the safety and efficacy of various ultrasound-guided carpal tunnel release procedures, and to compare their clinical outcomes with traditional open, mini-open, and endoscopic procedures.

## 2. Materials and Methods

PubMed, PubMed Central, and Scopus were searched. Studies were identified, screened, and assessed for eligibility against predefined inclusion and exclusion criteria, following PRISMA guidelines. This review asked which ultrasound-guided surgical techniques are used in carpal tunnel syndrome (CTS) and how safe they are, with the aim of identifying the most frequently reported approaches. The search covered the preceding 14 years.

Studies were eligible for inclusion if they reported an ultrasound-guided carpal tunnel release technique performed on human participants; reported at least one clinical, functional, electrophysiological or imaging outcome, or the occurrence of adverse events; enrolled more than one patient; and were published in English as full-text articles. Studies comparing an ultrasound-guided technique with open, mini-open, or endoscopic release were also eligible.

Studies were excluded if they were published in a language other than English; if the full text or abstract was inaccessible; if reporting of intervention characteristics or outcomes was incomplete to the extent that reliable data extraction was not possible; if the surgical technique was performed without ultrasound guidance; if they were single-case reports; or if they addressed subject matter outside of the objectives of this review. Studies reporting economic outcomes exclusively (cost-effectiveness and healthcare costs) were excluded because this review addressed the safety, efficacy, and technical characteristics of ultrasound-guided procedures rather than their cost-effectiveness; studies reporting clinical outcomes alongside cost data remained eligible. Access model was not a criterion—both open-access and subscription publications were eligible.

The database search was conducted between 7 December 2023 and 10 February 2024, in accordance with a predefined search strategy. The search strategy was based on combinations of the following keywords: “carpal tunnel syndrome,” “CTS,” “ultrasound-guided,” “carpal tunnel release,” “open carpal tunnel release,” “endoscopic carpal tunnel release,” “percutaneous ultrasound-guided carpal tunnel release,” “mini-open carpal tunnel release,” and “thread carpal tunnel release.” The same predefined search strategy was applied to all searched databases. Records retrieved from all sources were combined, and duplicates were removed. Titles and abstracts were screened, and the full texts of potentially eligible records were assessed against the eligibility criteria, independently and in duplicate, by two reviewers (M.P. and K.M.). Disagreements at each stage were resolved by discussion until consensus was reached; no third adjudicator was required.

The following items were recorded from each study: participant characteristics, the ultrasound-guided technique and any comparator procedure, access or incision size, anesthetic and procedural setting, number of patients and hands, duration of follow-up, outcome instruments used and reported results, adverse events, and disclosed funding and conflicts of interest. Where a study reported an outcome at several time points, the longest reported follow-up was extracted.

The methodological quality and risk of bias of the included studies were assessed using the Joanna Briggs Institute (JBI) Critical Appraisal Checklists corresponding to each study design (case series, cohort, randomized controlled trials, and quasi-experimental studies). Two reviewers (M.P. and K.M.) applied the checklists independently, and any discrepancies were resolved through discussion and consensus.

**Figure 1:**
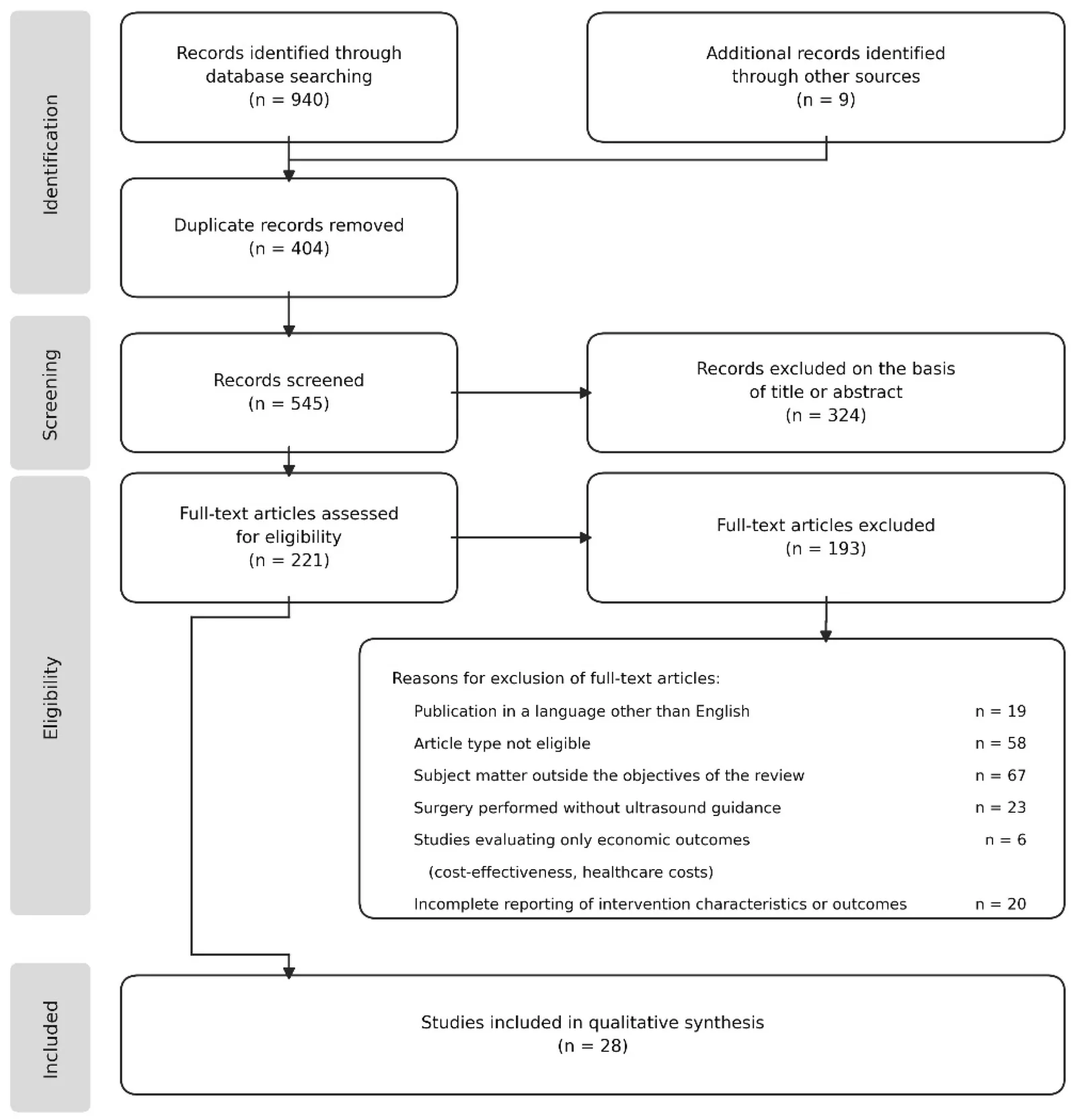
Flowchart of study selection.

The search identified 940 records through database searching and a further 9 through other sources. After removal of 404 duplicates, 545 records were screened based on title and abstract, of which 324 were excluded. Of the 221 full-text articles assessed for eligibility, 193 were excluded for the reasons detailed in Figure 1, leaving 28 studies for inclusion in the qualitative synthesis (Figure 1).

The following data were extracted from each study:

1. Type of procedure performed;
2. The number of patients included in the study and the number of hands operated on;
3. Length of follow-up of patients after surgery;
4. Clinical evaluation using instruments;
5. Side effects;
6. Conclusions of the study.

A detailed review of the articles was also carried out in terms of the following:

1. Trials of more than 100 patients;
2. The presence of a control group;
3. Same follow-up time for all patients participating in the study;
4. Potential conflict of interest;
5. Randomized controlled trial (RCT) type study;
6. Patient followed for more than 12 months;
7. Standardized patient assessment tools after surgery.

In total, 193 studies did not meet the inclusion criteria. The included studies varied considerably in participant characteristics, interventions, assessment methods, and reported outcomes, and no meta-analysis was undertaken. Results were instead summarized as a narrative synthesis. No quantitative synthesis was undertaken, and no meta-analysis was planned at the design stage, given the anticipated clinical and methodological heterogeneity of the eligible literature. No statistical analyses were therefore performed on aggregated data; the descriptive frequencies reported in the Results section were calculated directly from the extracted data. The methodological appraisal was conducted independently by two of the authors (M.P. and K.M.); no independent statistician was involved in the preparation of this review.

Additional methodological details and supporting materials are provided in the Supplementary Materials (Tables S1–S4) [26].

## 3. Results

### 3.1. Study Characteristics

In this systematic review, we included articles published in English in the last 14 years. A total of 28 studies were included. The largest group was case series (17 studies, 60.7%), indicating that the available evidence is primarily observational. A further four prospective cohort studies (14.3%), four randomized controlled trials (RCTs; 14.3%), two retrospective cohort studies (7.1%), and one quasi-experimental study (3.6%) were identified. Details of the included studies are given in Table 1.

The majority of patients undergoing surgery for carpal tunnel syndrome were women: women predominated in 22 studies and men in two, while four studies did not report participant sex. One study enrolled only women, aged 52–71 years, all homemakers, with idiopathic carpal tunnel syndrome; eligibility required thenar muscle weakness or intractable pain limiting activity [35]. Bilateral surgery was inconsistently reported—10 studies gave no information on it, and among the 18 that reported the number of hands operated on, the proportion ranged from 0% [20] to 70% [27].

### 3.2. Surgical Techniques and Complications

Across the included studies, sample sizes ranged from 14 to 152 participants and follow-up from 3 to 74 months. The most frequently reported ultrasound-guided approach was device-assisted carpal tunnel release (CTR-US; 11 studies), followed by thread carpal tunnel release (TCTR; five studies) and percutaneous carpal tunnel release (PCTR; five studies). Supplementary Table S2 compares the incidence of postoperative complications across all ultrasound-guided carpal tunnel release techniques evaluated in the 28 included studies—UMIU-CTR, TCTR, PCTR, CTR-US (using MicroKnife, SX-One, or MICROi-Blade devices), and MICTR (MANOS)—against reference methods: open carpal tunnel release (OCTR, mini-OCTR, and MOCTR) and endoscopic release (ECTR and MECTR). The main findings indicate that most ultrasound-guided techniques are characterized by a very low complication rate. No complications were reported for UMIU-CTR or for the comparative ECTR/MECTR technique. For TCTR and CTR-US, the rate was also low (approximately 0.5–0.8% and ≥0.5%, respectively), and the reported events were most often transient and resolved spontaneously. A higher rate was observed for PCTR (1.6%), largely attributable to a single study, while MICTR (MANOS) showed the highest incidence (8.9%), including persistent numbness, swelling, and the need for reoperation for incomplete ligament release. Overall, the results suggest that ultrasound-guided carpal tunnel release techniques are safe, and most reported complications are mild and transient. The supplement also includes Table S3, which presents the chronological development of techniques and devices used for ultrasound-guided carpal tunnel release between 2010 and 2024. The analysis shows a clear evolution from the first percutaneous techniques using a blade and guide wire (PCTR) to increasingly less invasive methods requiring only needle punctures, such as TCTR and UMIU-CTR. In subsequent years, dedicated devices for carpal tunnel release were developed, in particular the SX-One MicroKnife and MICROi-Blade systems, which were designed to increase the safety of the procedure by better protecting the median nerve and adjacent structures. At the same time, the diameter of the surgical access site decreased from approximately 4 mm to ≤1 mm, which helped minimize tissue trauma and accelerate patient recovery. The most recent publications from 2023 to 2024 no longer focus on the development of new devices, but rather on providing high-quality scientific evidence, including randomized controlled trials and long-term follow-up studies confirming the efficacy and safety of CTR-US techniques.

### 3.3. Clinical Outcomes

Outcome measurement varied widely across the included studies, and no single instrument was applied by all of them. Patient-reported symptom severity and function were most often assessed with the Boston Carpal Tunnel Questionnaire or its symptom-severity and functional-status subscales, including the Levine–Katz instrument from which it derives, in 19 of the 28 studies [16,17,18,20,21,30,31,32,33,34,35,36,37,38,40,41,42,43]. The DASH or QuickDASH was used in 10 studies [13,14,15,19,21,29,32,37,38,40], and pain was recorded on a visual analogue or numeric scale in eight [8,15,20,28,35,39,42,43]. Generic health-related quality of life was captured with the EQ-5D-5L in two studies [20,35] and hand-specific quality of life with the Michigan Hand Outcomes Questionnaire in 1 [20]. Objective measures were applied less consistently: grip or pinch strength in eight studies [8,13,14,16,29,33,34,42], two-point discrimination in six [8,13,16,28,34,39], Semmes–Weinstein monofilament testing in four [16,28,34,39], and electrodiagnostic assessment in six [8,11,28,33,39,43]. Imaging endpoints were reported in four studies, comprising magnetic resonance imaging [15,19] and sonographic measurement of median nerve cross-sectional area [41,43]. Patient satisfaction was recorded in six studies [20,21,32,33,38,40], and time to resumption of daily activities or work in five [8,14,28,35,42]. No study defined treatment success a priori against a common threshold, and no outcome was reported at a uniform time point across studies, so results are described below by direction of effect rather than by pooled magnitude.

All 28 studies reported improvement in symptom severity and hand function from baseline to final follow-up, and none reported failure of its primary outcome. Where the Boston questionnaire was used, both the symptom-severity and functional-status subscales improved in every study reporting them, with the earliest improvements described within one to two weeks of the procedure [38,40] and improvement sustained at the longest reported follow-up of 30 to 74 months [31]. Studies using the DASH or QuickDASH reported the same direction of change. This uniformity should be interpreted with the study designs in mind: 17 of the 28 studies were single-arm case series, in which improvement from baseline cannot be separated from patient selection, expectation, and natural history.

Six studies included a concurrent comparator arm and therefore permit direct comparison. Rojo-Manaute et al. reported faster functional recovery and fewer postoperative complications after UMIU-CTR than after mini-open release at 12 months [14], and Capa-Grasa et al. reported comparable outcomes for the ultra-minimally invasive technique against mini-open release at 3 months [13]. Nakamichi et al. found neurological recovery after percutaneous release equivalent to mini-open release, with earlier return to function, at 24 months [28]. In the largest randomized comparison, Eberlin et al. reported clinical improvement in symptoms, function, and quality of life within 3 months in both the CTR-US and mini-open groups, with rapid return to work in each [35]. Akhoondinasab et al. found the effectiveness of thread release and open release comparable at 3 months [39], and Chen et al. reported shorter operative and hospitalization times and earlier return to normal activity after modified endoscopic release than after open release at 41 months [8]. Across these comparisons, the consistent finding is equivalence in symptomatic and functional outcome, with any advantage of the ultrasound-guided approaches confined to the speed of early recovery; no study demonstrated superiority of a CTR-US technique on a symptom or function endpoint at final follow-up.

Objective and imaging endpoints were broadly concordant with the patient-reported findings. Grip and pinch strength improved in the studies reporting them [8,13,14,16,29,33,34,42], and sensory testing showed improvement or no deterioration [8,13,16,28,34,39]. Electrodiagnostic parameters improved where measured [11,33,39,43]. Sonographic cross-sectional area of the median nerve decreased after release in one series as early as two to four weeks postoperatively [41], and magnetic resonance imaging confirmed division of the transverse carpal ligament with anatomical decompression comparable to that achieved by conventional surgery [15,19]. These findings support the anatomical adequacy of ultrasound-guided division but do not, in themselves, establish clinical equivalence with open or endoscopic release.

Follow-up ranged from 3 to 74 months. Fourteen studies followed patients for at least 12 months [8,11,15,16,17,21,28,29,31,34,36,37,40], while the remainder reported outcomes at 3 to 6 months only, and follow-up duration was uniform across all participants in only four studies. One cohort was restricted to patients receiving hemodialysis, a population in which outcomes after carpal tunnel release are less well characterized [34]. Complications and adverse events are reported by technique in Section 3.2 and in Supplementary Table S2. Study-level details for all outcomes, including the instruments used and the authors’ conclusions, are given in Table 2.

The most commonly used clinical assessment scale was the Boston Carpal Tunnel Questionnaire (BCTQ) or its subscales (BCTQ-SSS/FSS), which were used in at least 13 studies. The second most commonly used method was the QuickDASH/DASH (9), used to assess upper limb function and the degree of disability. Objective functional measurements were also frequently used, such as grip strength, the two-point discrimination test, the Semmes–Weinstein test, EMG/nerve conduction studies, and pain scales (VAS and Numeric Pain Scale). Specialized tools, such as the EQ-5D-5L, MHQ (Michigan Hand Outcomes Questionnaire), Levine-Katz Questionnaire, Modified Mayo Wrist Score, Hamada Classification, and MRI evaluation, were used least frequently.

The primary outcomes were the severity of carpal tunnel syndrome symptoms, improvement in hand function, pain reduction, grip strength, patient satisfaction, time to return to daily activities or work, and surgical safety assessed based on the number and nature of complications. In some studies, imaging findings (MRI, ultrasound, and median nerve cross-sectional area) and electrophysiological parameters were also evaluated to objectively confirm the effectiveness of median nerve decompression.

Across the included studies, sample sizes ranged from 14 to 152 participants, with follow-up from 3 to 74 months. The most frequently reported ultrasound-guided approach was device-assisted carpal tunnel release with ultrasound guidance (CTR-US; 11 studies), followed by thread carpal tunnel release (TCTR; five studies) and percutaneous carpal tunnel release (PCTR; 5 studies). One cohort included only hemodialysis patients. Study-level procedural details are summarized in Table 2.

Adverse events were described in 14 of 28 studies and were predominantly minor and transient (e.g., local swelling, temporary numbness or neuritis, and superficial infection). Serious neurovascular injury and reoperation due to incomplete release were rare and reported only in isolated cases (Table 2).

To assess the level of transparency of the data provided and the methodological quality of the studies analyzed, we conducted an additional comparison, the results of which are presented in Table S1 (Supplementary Materials). We assessed the presence of the following elements: the use of standard clinical assessment tools for postoperative patients, follow-up of patients for at least 12 months after surgery, randomized controlled trial (RCT) design, disclosure of potential conflicts of interest, uniform follow-up duration for all participants, the presence of a control group, and a sample size exceeding 100 patients. Each of the analyzed parameters was assigned 1 point. A higher score indicated greater completeness of the reported data and higher study reliability.

In 21 trials, the number of patients was less than 100. Only four trials had a control group. Achieving a uniform follow-up duration was difficult for most studies, as patients were frequently lost to follow-up or could not be contacted for extended periods after surgery. Only 4 trials managed to follow up all patients for the same length of time after surgery. A potential conflict of interest was identified in 17 of the included studies. This included financial and professional relationships between the authors and the manufacturers of the devices used during surgery. Patients were followed up one year after surgery in 11 trials. Standardized and validated tools for the clinical assessment of patients after surgery were available in 24 trials. None of the eligible studies in the systematic review achieved the maximum score of 7. One study achieved a score of 5 [14], followed by three studies with a score of 4 [31,35,39].

### 3.4. Methodological Quality

An assessment of methodological quality and the risk of systematic error was conducted using the appropriate JBI checklists, and the results are presented in Table 3. The Joanna Briggs Institute (JBI) checklists, adapted to the type of study, were used to assess methodological quality. For case series, a tool comprising 10 assessment criteria was used; for cohort studies, a checklist containing 11 criteria was used; for quasi-experimental studies, a checklist consisting of nine criteria was used; and for RCTs, a tool comprising 13 assessment criteria was used. Due to the varying number of criteria across the different JBI tools, the results were presented as the percentage of positive responses, which allowed for a direct comparison of the methodological quality of all included studies.

To standardize the presentation of results, each criterion rated as “yes” was assigned 1 point, while responses of “no,” “unclear,” and “not applicable” received 0 points. The number of criteria met was then summed and converted to a percentage of the maximum possible score for the given tool. The result was presented as “JBI criteria fulfilled (%).”

The results of the methodological quality assessment indicate a generally high level of reliability among the included studies. The percentage of JBI criteria met ranged from 60% to 100%, with most publications scoring at least 80%. Five studies met all the criteria of the relevant JBI tools. It is worth noting that several authors defined their studies as cohort studies; however, a detailed methodological analysis revealed the absence of key characteristics of cohort studies, such as the presence of an appropriate comparison group. Consequently, these studies were classified as case series and assessed using the JBI checklist dedicated to case series.

## 4. Discussion

Across the 28 included studies, ultrasound-guided carpal tunnel release (CTR-US) achieved symptomatic and functional improvement comparable to open (OCTR) and endoscopic (ECTR) release, with rapid resumption of daily activity and few reported adverse events. The evidence supporting this is weaker than the consistency of the findings suggests. Most of the literature consists of single-arm observational series of modest size. A small number of research groups produced much of it, often with financial links to the manufacturers of the devices they evaluated, and follow-up was short and unevenly applied. These constraints limit how firmly the advantages of CTR-US can be asserted.

### 4.1. Study Design and the Level of the Available Evidence

Of the 28 included studies, only four were randomized [8,14,35,39], and only three of these compared an ultrasound-guided technique with an open procedure [14,35,39]; the fourth compared modified endoscopic with open release and was retained as comparator context [8]. Twenty-one studies enrolled fewer than 100 patients, only four included a control group, and follow-up was uniform across all participants in only three. The largest randomized comparison of CTR-US with mini-open release reports outcomes at 3 months [35], and the longest randomized follow-up of any ultrasound-guided technique is 12 months [13]. All remaining comparisons with OCTR or ECTR are therefore indirect, drawn from separate single-arm cohorts differing in case mix, severity thresholds, operator experience, and outcome instruments.

Two consequences follow. Single-arm series cannot separate the effect of the technique from patient selection, expectation, and natural history. The uniformly favorable conclusions of the included cohorts (Table 2) are compatible with a genuine treatment effect; they are equally compatible with the selection of favorable candidates for a novel procedure. The Joanna Briggs Institute appraisal (Table 3) is also easy to over-read. It measures reporting completeness within a given design, not comparative validity—a case series scoring 100% of JBI criteria remains a case series and offers weaker comparative evidence than a randomized trial scoring 77%.

### 4.2. Heterogeneity of Interventions and of Outcome Measurement

CTR-US is not a single procedure. The included studies applied at least five distinct approaches: percutaneous release with a hook or retractable blade, thread transection (TCTR), ultra-minimally invasive release through a ≤1 mm portal (UMIU-CTR), balloon-assisted device release, and needle-mounted blade techniques. Skin openings ranged from 0.4 mm to approximately 10 mm, and settings from the operating room to the office under WALANT (Table 2). A single label flattens these differences. Because the approaches divide the transverse carpal ligament by different mechanisms, they carry different plausible failure modes, and any claim about the safety of CTR-US is really a claim about a family of procedures rather than one.

Outcome measurement was equally heterogeneous. Symptom and function endpoints included the BCTQ (SSS and FSS), the Levine–Katz questionnaire, QuickDASH/DASH, the Michigan Hand Questionnaire, CTS-6, VAS and numeric pain scales, and EQ-5D-5L, supplemented in various combinations by grip strength, two-point discrimination, Semmes–Weinstein testing, electrodiagnostic parameters, sonographic median nerve cross-sectional area, and MRI [13,14,28,34,37]. No outcome was reported by every study, and none defined success a priori using a common threshold. Return to work—arguably the endpoint most relevant to office-based release—was reported in a minority of cohorts. Follow-up ranged from 3 to 74 months, and only 10 studies reached 12 months. Given interventions, endpoints, and follow-up intervals that differ this widely, we did not attempt a quantitative synthesis.

### 4.3. Comparison with Open and Endoscopic Release

Within these constraints, the comparative signal is narrow. In the multicenter randomized trial by Eberlin et al., CTR-US and mini-open release produced comparable improvements in symptoms, function, and quality of life at 3 months, with rapid return to activity in both arms; CTR-US was not superior on the primary clinical endpoints [35]. Rojo-Manaute et al. reported faster functional recovery after UMIU-CTR than after mini-open release at 12 months [14]. Registry data set a benchmark that none of these cohorts can approach. Across more than 850,000 open decompressions in England, Lane et al. recorded serious complications in 0.082% and reoperation in 3.42% [44]—figures that no CTR-US series in this review is large enough to match or refute. Against ECTR, the case for CTR-US rests on avoiding general anesthesia and operating-room resources, and on continuous visualization of neurovascular structures; superiority of clinical outcome has not been shown [7,10]. Nicholas et al. and Chappell et al. confirm that ultrasound-guided division achieves anatomical decompression comparable to conventional release [19,41]. Anatomical adequacy, however, does not by itself establish clinical equivalence.

### 4.4. Safety Reporting

Fourteen of the 28 studies reported no intraoperative or postoperative complications. This is not the same as evidence of safety. Few studies prespecified adverse-event definitions, and none described active surveillance or independent adjudication; in most, ascertainment was limited to events presenting at routine follow-up. The distribution of reported events matters here. The highest complication rate in the review—paresthesia in the median nerve distribution in 26.3% of hands—comes from a small independent series [43], while several larger cohorts run by developers or early adopters of specific devices report none. Isolated serious events did occur: nerve contusion [20], iatrogenic partial nerve injury requiring neurolysis [35], postoperative infection and compartment syndrome requiring reintervention [21], and complex regional pain syndrome [14]. With cohorts of 14 to 152 patients, these data are compatible with a true rate of serious complications anywhere within the range reported for open surgery and cannot establish that it is lower.

### 4.5. Learning Curve and Operator Dependence

CTR-US replaces direct visual control with sonographic interpretation, so its safety depends on operator competence in a way open release does not. Several included studies are explicitly reports of early experience [15,19], or of implementing an established technique in a new center [14]. The developers of TCTR acknowledged a learning curve from the outset and recommended formal ultrasound training before clinical use [17]. Eberlin et al. went further, reporting that their trial investigators were substantially more experienced with mini-open release than with CTR-US—a factor that the authors accepted may have biased the comparison [35]. Yet no included study defined a competency threshold. None estimated the case volume required for proficiency, and none analyzed outcomes by operator experience. The reported results therefore describe what motivated early adopters achieve; whether unselected surgeons achieve the same is untested.

### 4.6. Concentration of the Evidence Base, Industry Involvement and Publication Bias

The literature on CTR-US is generated by a small number of groups. At least 15 of the 28 included studies originate from six research teams. The Guo group developed TCTR [17,18], and the Rojo-Manaute and Capa-Grasa group developed UMIU-CTR and co-authored its subsequent implementation study [13,14,15]. Two further teams—Chern and Jou in Taiwan [16,34], and the Nazarian group in Philadelphia [21,29]—account for four more. The Joseph, Beckman, and Leiby group contributes four [31,38,40,41], and two industry-funded multicenter studies of the same device platform contribute the remainder [20,35]. Several techniques are therefore supported almost entirely by evidence from the teams that devised them.

Financial relationships with device manufacturers were disclosed in 17 of the 28 included studies, and several were substantial. The multicenter randomized trial by Eberlin et al. and the ROBUST trial were both funded by the device manufacturer, with sponsor involvement in trial design and, in ROBUST, in data collection and interpretation [20,35]. Authors at one center disclosed employment by the manufacturer, consultancy, and equity holdings [38,40]. Another series disclosed a royalty agreement and receipt of the first devices free of charge [42], and the originating TCTR study disclosed a financial interest in the instrumentation [17]. These disclosures are appropriate, and none of them invalidates a finding. What they establish is a consistent direction of potential bias: sponsor involvement in design and interpretation is associated with more favorable reported outcomes in device research generally [43].

These features make publication bias difficult to exclude and impossible to quantify. Nearly all included studies report favorable conclusions, and none of the ultrasound-guided cohorts was prospectively registered. Small unfavorable early-experience series are unlikely to reach publication in the first place. Without a meta-analysis, funnel-plot and other statistical assessments of small-study effects are unavailable, and our restriction to English-language, full-text publications may have narrowed the retrieved evidence further. The defensible reading is a modest one. CTR-US is feasible and performs acceptably in the hands of experienced—and often financially interested—investigators. Whether it is as safe and durable as open release in routine practice is a question that independent, adequately powered, prospectively registered trials have yet to answer.

### 4.7. Implications for Research

Several priorities follow from the deficiencies identified here. The first is independent, prospectively registered randomized trials comparing a defined CTR-US technique with mini-open release, powered for complications and not for symptom scores alone. The second is a core outcome set built around the BCTQ and QuickDASH, with prespecified success thresholds and standardized follow-up at 3, 12, and 24 months. Beyond trial design, three reporting requirements matter: operator experience and learning-curve effects should be stated explicitly, adverse-event ascertainment should be systematic and adjudicated, and comparative studies should be conducted by groups with no financial interest in the devices evaluated. Higher-risk populations, including patients on hemodialysis [34], remain particularly under-studied.

### 4.8. Limitations

Several limitations should be noted. Most included studies were observational single-center cohorts and case series, small, and methodologically heterogeneous, thus limiting direct comparison between techniques. Follow-up was often short, and few cohorts extended beyond 12 months. The included studies differed in regard to treatment indications, patient characteristics, surgical technique, outcome instruments, and reported endpoints; meta-analysis was therefore not undertaken, and results are presented as a narrative synthesis. Differences in operator experience and in learning curves for newer techniques may also have influenced reported outcomes.

This review was not prospectively registered in PROSPERO. The search covered PubMed, PubMed Central, and Scopus only; closed in February 2024; and was restricted to English-language full-text publications. Relevant studies may therefore have been missed. Many included studies evaluated commercially developed devices, and a potential conflict of interest could not be excluded in a substantial proportion of them. These studies generally reported favorable outcomes, but the available data do not permit any assessment of how far commercial involvement shaped them.

Within these constraints, this review sets out the evidence currently available across ultrasound-guided and conventional approaches, and identifies where independent comparative research is most needed.

## 5. Conclusions

Ultrasound-guided carpal tunnel release is a promising minimally invasive alternative to open and endoscopic techniques. Reported series describe rapid recovery and minimal soft-tissue disruption, and the anatomical adequacy of release appears comparable to conventional surgery. The evidence supporting these observations is weak. It rests largely on small single-arm cohorts, produced by a limited number of groups and frequently with industry involvement, with short follow-up and no standardized definition of success. Newer approaches, such as TCTR and UMIU-CTR, extend what can be done in an office setting, but their safety and durability are not yet established. Independent randomized trials, adequately powered for complications and reporting funding sources transparently, are needed before CTR-US can be regarded as an established alternative to open or endoscopic release. Standardized technique descriptions and a core outcome set would make the necessary comparisons possible.

## Figures and Tables

**Table 1:** Study details.

Study/Characteristic	Sex [%]	Two-Handed Operation [%]	Type of Study
Krogh et al. (2023), Denmark [15].	N/d	N/d	Prospective study, case series
Petrover et al. (2017), France [27].	69.8% W 30.2% M	N/d	Prospective study, cohort studies
Rojo-Manaute et al. (2016), Spain [14].	UMIU-CTR 58.6% W 41.4% M BMCTR 63% W 37% M	N/d	RCT
Nakamichi et al. (2010), Japan [28].	100% W	N/d	Prospective comparative study, case series
McShane et al. (2012), USA [29].	89% W 11% M	N/d	Retrospective study, case series
Guo et al. (2014), USA [18].	60% W 40% M	70%	Pilot study, case series
McCormack et al. (2012), USA [30].	69.23% W 30.77% M	7.69%	Retrospective study, case series
Guo et al. (2017), USA [17].	66.38% W 33.62% M	37.07%	Prospective study, case series
Cano et al. (2024), USA [31].	66.7% W 33.3% M	58.8%	Retrospective cohort study
Capa-Grasa et al. (2014), Spain [13].	N/d	N/d	Pilot study, case series
Henning et al. (2018), USA [32].	N/d	N/d	Prospective clinical observation, case series
Burnham et al. (2021), Canada [33].	55% W 45% M	0%	Controlled prospective study, quasi-experimental studies
Wang et al. (2019), Taiwan [34].	67% W 33% M	40%	Prospective case series, case series
Eberlin et al. (2023), USA [35].	CTR-US: 63.8% W 36.2% M MOCTR 75% W 25% M	N/d	RCT
Kim et al. (2024), Republic of Korea [36].	68.45% W 31.54% M	10.53%	Retrospective cohort study
Bergum et al. (2022), USA [37].	57% W 43% M	33%	Prospective case series
Li et al. (2019), China [11].	67.7% W 32.3% M	41.9%	Prospective observational study
Joseph et al. (2020), USA [38].	59.1%W 40.9% M	59.1%	Retrospective, prospective, case series
Akhoondinasab et al. (2024), Iran [39].	95% W 5% M	N/d	RCT
Chen et al. (2021), China [8].	63.8% W 36.2% M	N/d	RCT
Pistorio et al. (2024), USA [20].	51.7% W 48.3%M	57.7%	Prospective observational study, case series
Nicholas et al. (2024), USA [19].	68% W 32% M	47%	Retrospective observational study, case series
Kamel et al. (2020), USA [21].	54% W 46% M	33%	Retrospective observational study, cohort study
Leiby et al. (2022), USA [40].	N/d	61.7%	Prospective cohort study
Chappell et al. (2020), USA [41].	30% W 70% M	60.9%	Retrospective, prospective, case series
Hebbard et al. (2021), Australia [42].	46.36% W 53.64% M	50.91%	Case series
Chern et al. (2015), Taiwan [16].	77.5% W 22.5% M	17.5%	Case series
Luanchumroen et al. (2019), Thailand [43].	87.5% W 12.5% M	6.25%	Prospective cohort study, case series

Abbreviations: W—women; M—men; RCT—randomized controlled trial; N/d—no data.

**Table 2:** Details of the trials included in the review.

No.	Name	Procedure	Number of Patients	Months	Clinical Assessment	Side Effects	Conclusions
1	Krogh et al. (2023), Denmark [15].	UMIU-CTR (1 mm)	10 patients—10 hands	12	DASH, CTS-6, Palmar Pain Scale, and MRI	No intra- or postoperative complications were reported.	UMIU-CTR is a safe and effective technique. It has been shown to significant improvement in symptom reduction, improvement in performance, and persistence of positive effects throughout the follow-up period.
2	Petrover et al. (2017), France [27].	PCTR (2.0–5.0 mm)	129 patients—129 hands	6	Rupture of the transverse carpal ligament, decompression of the nerve, and reported symptoms	No intra- or postoperative complications were reported.	The ultrasound-guided PCTR procedure is effective in relieving patient-reported symptoms and is safe and feasible.
3	Rojo-Manaute, et al. (2016), Spain [14].	UMIU-CTR (1 mm)/ MOCTR (2 cm)	92 patients—92 hands UMIU-CTR—46 BMCTR—46	12	QuickDASH, shoulder, and hand; grip strength; time of cessation of oral analgesics; complete flexion-extension of the wrist; resolution of paresthesia; and return to normal daily activities and work	No complications were reported in the UMIU-CTR patient group. In patients in the MOCTR group, the following were reported: symptoms of complex regional pain syndrome and superficial infection, but these were isolated cases.	UMIU-CTR using ultrasound provides faster functional recovery and lower incidence of postoperative complications compared to blind mini-open carpal tunnel release.
4	Nakamichi et al. (2010), Japan [28].	PCTR (4 mm)/MOCTR (1–1.5 cm)	65 patients—74 hands PCTR- 35 hands 29 patients MOCTR—39 hands 36 patients	24	Medical history, muscle strength, feeling, pain (0–3), Phalen test, reverse Phalen test, Tinel’s sign, EMG, two-point discrimination test, Semmes–Weinstein fiber test, and scar sensitivity	No intra- or postoperative complications were reported.	PCTR provides the same neurological recovery as mini-OCTR. PCTR provides fewer postoperative complications and an earlier return to function and satisfaction.
5	McShane et al. (2012), USA [29].	PCTR (0.5–1.2 mm)	17 patients—17 hands	25	DASH, Phalen test, Tinel test, wrist compression test, assessment of glenohumeral muscle atrophy, thumb dehiscence, upper limb tension test, hand grip strength, and symptoms and functional impairment questionnaire	No intra- or postoperative complications were reported.	PCTR treatment leads to a reduction in symptoms and improvement in function, reducing pressure on the median nerve and, thus, resulting in a high level of satisfaction among patients. The method can be an effective alternative to traditional surgical methods.
6	Guo et al. (2015), USA [18].	TCTR (0.5–1.2 mm)	20 patients, 34 hands	3	The Levine–Katz questionnaire	One case of swelling of the wrist and hand and restriction of finger flexion. After 3 days, the symptoms disappeared. They cannot be clearly linked to the TCTR procedure.	The TCTR technique is minimally invasive, safe, effective, and well tolerated by patients.
7.	McCormack et al. (2012), USA [30].	MICTR with MANOS (2.1 mm)	52 patients, 56 hands	3	Validated questionnaire on symptom severity and functional status	Complications: Redness and swelling at the insertion site treated with antibiotics (2), persistent swelling for up to 3 months (1), numbness persisting for up to 3 months (1), and reoperation due to incomplete release of the transverse ligament (1).	Despite the occurrence of complications, surgery using the MANOS device appears to be safe and effective.
8.	Guo et al. (2017), USA [17].	TCTR (0.4–1.2 mm)	116 patients, 159 hands	12	BCTQ	No intra- or postoperative complications were reported.	The modified TCTR technique is safe and effective for the treatment of CTS. The technique is characterized by a short recovery time, minimal pain, and small scars
9.	Cano et al. (2024), USA [31].	CTR-US (>5 mm) MicroKnife	102 patients, 162 hands	30–74 Average time: 46	BCTQ, Symptom Scale and Functional Status Scale	Transient sign of nerve inflammation (1).	The CTR-US technique is an effective and safe method of treating CTS, providing long-term symptom relief and improved hand function.
10.	Capa-Grasa et al. (2014), Spain [13].	MOCTR (~2 cm), UMIS (≤1 mm)	40 patients—40 hands	3	QuickDASH, grip strength rate, two-point discrimination, and recovery time	No intra- or postoperative complications were reported.	The Ultra-MIS technique can be safe and effective for patients with CTS.
11.	Henning et al. (2018), USA [32].	CTR-US (>5 mm) MicroKnife	14 patients—19 hands	3	QuickDASH, BCTQ, improvement in symptoms, and satisfaction with the results of the treatment	No intra- or postoperative complications were reported.	The US-CTR technique using the MicroKnife device is a promising, safe, and effective technique for the treatment of CTS.
12.	Burnham et al. (2021) Canada [33].	TCTR (0.4–1.2 mm)	20 patients, 20 hands	6	BCTQ, satisfaction survey, nerve conduction, and measurement of grip strength and compression	No intra- or postoperative complications were reported.	The TCTR technique appears to be safe and well tolerated by patients, as evidenced by high patient satisfaction after surgery.
13.	Wang et al. (2019), Taiwan [34].	PCTR (0.8–1.2 mm)	84 patients—113 hands All patients were hemodialyzed	24	CTS-SSS, CTS-FSS, two-point discrimination test, Semmes–Weinstein monofilament test, grip force measurement, three-point squeeze strength measurement, symptom improvement assessments, and pain and numbness assessment	No intra- or postoperative complications were reported.	The PCTR technique is less invasive than OCTR and ECTR, as evidenced by the reduced risk of complications and shorter recovery time in CTS patients.
14.	Eberlin et al. (2023), USA [35].	CTR-US (6 ± 2 mm), MOCTR (22 ± 7 mm)	122 patients—122 hands CTR-US—101, MOCTR—48	3	BCTQ, Symptom S Numeric Pain Scale, and EQ-5D-5 L	Noted 3 complications CTR-US (2). Common palmar nerve injury MOCTR (1). Wound dehiscence.	Both treatment groups reported rapid return to work and activity, and clinical improvement in symptoms, function, and quality of life within 3 months.
15.	Kim et al. (2024), Republic of Korea [36].	TCTR (0.4–1.2 mm)	152 patients—168 hands	24	BCTQ-SSS and BCTQ-FSS	1 complication—swelling of the wrist (iatrogenic partial removal of the accessory adductor muscle of the little finger); symptoms resolved spontaneously after 2 weeks.	The TCTR technique is a safe and effective treatment method.
16.	Bergum et al. (2022), USA [37].	OCTR (<5 mm)	88 patients, 123 hands	12	BCTQ and QDASH	One case—Complex Regional Pain Syndrome (CRPS).	OCTR using ultrasound is safe and effective, and the technique is well tolerated by patients.
17.	Li et al. (2019), China [11].	ECTR (1–1.5 cm)	31 patients—44 hands	12	Gelberman scale and electrodiagnostic test (EDT)	No intra- or postoperative complications were reported.	The neuroanatomical parameters of the median nerve gradually improve after endoscopic carpal tunnel release (ECTR) at one-year follow-up.
18.	Joseph et al. (2020), USA [38].	CTR-US (>5 mm)	22 patients—35 hands	3	QDASH, BCTQ, and global patient satisfaction	No intra- or postoperative complications were reported.	USCTR is a safe and effective procedure that can be performed by an experienced sports medicine physician, and usually results in significant improvements within the first 2 weeks after the procedure.
19.	Akhoondinasab et al. (2024), Iran [39].	OCTR (6 cm), TCTR (1.5 cm)	20 patients—20 hands	3	VAS, monofilament test, two-point discrimination test, modified Mayo scale for the wrist Nerve conduction study and electromyography, and postoperative scar lengths	Pillar pain occurred (resolved spontaneously after 6 months).	The effectiveness of both techniques is comparable. TCTR may be a safe and effective alternative to OCTR in the treatment of carpal tunnel syndrome.
20.	Chen et al. (2021), China [8].	MECTR (1 cm)/OCTR (6 cm)	94 patients—94 hands	41	Hamada classification, EMG, Kelly grading, VAS, two-point discrimination test, duration of hospitalization, duration of surgery, time to return to normal work, and grip and compression strength	In one case in the OCTR group, there was a partial recurrence of symptoms after 3.5 months that resolved after an injection of triamcinolone acetonide.	MECTR provides a faster recovery: patients undergoing the MECTR technique had shorter surgery and hospitalization times and a faster return to normal life compared to patients in the OCTR group.
21.	Pistorio et al. (2024), USA [20].	CTR-US (3–8 mm) MicroKnife	149 patients—226 hands	6	BCTQ-SSS, BCTQ-FSS, MHQ, Numeric Pain Scale, EQ-5D-5L, satisfaction with the treatment, and side effects	One case—nerve contusion with a small suprascapular lesion on the ulnar side of the median nerve, with no evidence of damage to the nerve bundles. After neurolysis and wrapping of the nerve, symptoms resolved within 7 weeks, and median nerve function was fully restored.	CTR-US performed in an office setting is safe and effective.
22.	Nicholas et al. (2023), USA [19].	CTR-US (4–5 mm) MikroKnife	65 patients—96 hands	6	QuickDASH; BCTQ; and MRI evaluation—transverse carpal ligament (TCL) status. Median nerve diameter, median nerve signal intensity, and median nerve and carpal tunnel flattening ratio	No intra- or postoperative complications were reported.	CTR-US can be as effective as traditional surgical techniques in treating carpal tunnel syndrome, while offering faster recovery and lower risk of complications.
23.	Kamel et al. (2020), USA [21].	CTR-US (4–5 mm) SX-One MicroKnife	46 patients—61 hands	19	QDASH, BCTSQ-SS, BCTSQ-FS, and satisfaction with the treatment	Two cases of complications were reported: postoperative infection—methicillin-resistant Staphylococcus aureus (MRSA) syndrome.	Ultrasound-guided carpal tunnel release is effective in reducing pain and improving hand function in patients with CTS
24.	Leiby et al. (2022), USA [40].	CTR-US < 5 mm	47 patients—76 hands	12	BCTQ-SSS, BCTQ-FSS, QDASH, and satisfaction with the treatment	No intra- or postoperative complications were reported.	CTR-US is a safe and effective procedure that produces significant improvements in as little as 1–2 weeks after treatment, and these effects last up to a year.
25.	Chappell et al. (2020), USA [41].	CTR-US (4–5 mm)	23 patients—37 hands	3	BCTQ and CSA measurements of the median nerve	No intra- or postoperative complications were reported.	Microinvasive CTR-US leads to a reduction in median nerve CSA within 6–10 weeks after the procedure. A reduction in CSA was observed as early as 2–4 weeks after surgery, indicating a rapid resolution of median nerve edema.
26.	Hebbard et al. (2021), Australia [42].	CTR-US, (~10 mm) MICROi-Blade	110 patients—166 hands	6	BCTQ, handshake strength, average pain level after 24 h, pain-medication requirements, and average time to return to previous life and work	The following complications were reported: transient finger numbness, swelling, and discomfort in the carpal tunnel area.	CTR-US is an effective, safe, and well-tolerated treatment method with rapid results and minimal complications.
27.	Chern et al. (2015), Taiwan [16].	CTR-US (2 mm)	80 patients—91 hands	12	CTS-SSS, CTS-FSS, two-point discrimination test, Semmes–Weinstein test, and handshake strength. Thumb and finger grip strength.	No intra- or postoperative complications were reported.	CTR-US is a safe and effective treatment for carpal tunnel syndrome.
28.	Luanchumroen et al. (2019), Thailand [43].	PCTR (1.2–1.6 mm)	16 patients—19 hands	6	BCTQ, measurement of the cross-sectional area of the median nerve (CSA), EMG of the median nerve, and VAS	5 cases (26.31%) of paresthesia along the course of the median nerve were reported, and they resolved spontaneously within 1–2 week.	PCTR is an effective alternative to traditional surgical treatments for carpal tunnel syndrome. It is a minimally invasive procedure that is characterized by rapid wound healing, less trauma to surrounding tissues, and a high level of patient satisfaction.

Abbreviations: Techniques: MICTR—minimally invasive carpal tunnel release; PCTR—percutaneous carpal tunnel release; CTR-US—carpal tunnel release with ultrasound guidance; MOCTR—mini-open carpal tunnel release; UMIU-CTR—ultra-minimally invasive ultrasound-guided carpal tunnel release; TCTR—ultrasound-guided thread carpal tunnel release; OCTR—open carpal tunnel release; MECTR—modified endoscopic carpal tunnel release; ECTR—endoscopic carpal tunnel release; UMIS—ultra-minimally invasive. Scale: MHQ—Michigan Hand Questionnaire; EQ-5D-5L—EuroQoL-5 Dimension 5-Level; VAS—visual analogue scale; CSA—cross-sectional area; CTS-SSS—Carpal Tunnel Questionnaire Symptom Specific Scale; CTS-FSS—Carpal Tunnel Questionnaire Function Severity Scale; QDASH—The Disabilities of the Arm, Shoulder, and Hand Questionnaire; BCTQ—Boston Carpal Tunnel Syndrome Questionnaire; BCTQ-SSS—Boston Carpal Tunnel Syndrome Questionnaire Symptom Severity Scale; BCTQ-FSS—Boston Carpal Tunnel Syndrome Questionnaire function Severity Scale; TCL—transverse carpal ligament; EMG—electromyography; DASH—Disabilities of the Arm, Shoulder, and Hand; MRI—magnetic resonance imaging; CTS-6—Carpal Tunnel Syndrome 6.

**Table 3:** Quality assessment of included studies according to Joanna Briggs Institute (JBI) critical appraisal tools.

Author	Type of Study	JBI Checklist	JBI Criteria Fulfilled (%)
Krogh et al. (2023), Denmark [15].	Case series	Case series	100%
Petrover et al. (2017), France [27].	Prospective study	Cohort studies	90%
Rojo-Manaute et al. (2016), Spain [14].	RCT	Randomized controlled trials	76.92%
Nakamichi et al. (2010), Japan [28].	Prospective comparative study	Cohort studies	63.63%
McShane et al. (2012), USA [29].	Retrospective study, case series	Case series	60%
Guo et al. (2015), USA [18].	Pilot study	Case series	60%
McCormack et al. (2012), USA [30].	Retrospective study, case series	Case series	80%
Guo et al. (2017), USA [17].	Prospective study, case series	Case series	60%
Cano et al. (2024), USA [31].	Retrospective cohort study	Cohort studies	72.72%
Capa-Grasa et al. (2014), Spain [13].	Pilot study	Randomized controlled trials	84.61%
Henning et al. (2018), USA [32].	Prospective clinical observation, case series	Case series	80%
Burnham et al. (2021), Canada [33].	Controlled prospective study	Quasi-experimental studies	100%
Wang et al. (2014), Taiwan [34].	Prospective case series	Case series	100%
Eberlin et al. (2023), USA [35].	RCT	Randomized controlled trials	76.92%
Kim et al. (2024), Republic of Korea [36].	Retrospective cohort study	Cohort studies	81.81%
Bergum et al. (2022), USA [37].	Prospective case series	Case series	70%
Li et al. (2019), China [11].	Prospective observational study, case series	Case series	80%
Joseph et al. (2020), USA [38].	Retrospective, prospective, case series	Case series	90%
Akhoondinasab et al. (2024), Iran [39].	RCT	Randomized controlled trials	69.23%
Chen et al. (2021), China [8].	RCT	Randomized controlled trials	61.53%
Pistorio et al. (2024), USA [20].	Prospective observational study, case series	Case series	100%
Nicholas et al. (2024), USA [19].	Retrospective observational study, case series	Case series	90%
Kamel et al. (2020), USA [21].	Retrospective observational study	Cohort studies	90%
Leiby et al. (2022), USA [40].	Prospective cohort study	Cohort studies	90%
Chappell et al. (2020), USA [41].	Retrospective, prospective	Cohort studies	90%
Hebbard et al. (2021), Australia [42].	Case series	Case series	90%
Chern et al. (2015), Taiwan [16].	Case series	Case series	100%
Luanchumroen et al. (2019), Thailand [43].	Prospective cohort study, case series	Case series	90%

## Data Availability

All data are available in the manuscript.

## Supplementary Materials

The following supporting information can be downloaded at https://www.mdpi.com/article/10.3390/jcm15176675/s1, Table S1. Details of study standards; Table S2. Complication rate by ultrasound-guided carpal tunnel release technique; Table S3. Timeline of the evolution of ultrasound-guided carpal tunnel release techniques and devices; Table S4. PRISMA 2020 Checklist.
