# Supplementary material for: Safety and Effectiveness of Ultrasound-Guided Carpal Tunnel Release: A Systematic Review
Source: J Clin Med. 2026 Aug 28;15(17):6675. doi: 10.3390/jcm15176675 (PMC13566160; doi:10.3390/jcm15176675)
Supplement: Supplementary file 1 [file jcm-15-06675-s001.zip › Table S1. Details of study standards.pdf]

Table S1. Details of study standards.

Study/Characteristic	Sample of over 100 patients	Control group	Same observation time for all patients	No potential conflict of interest	RCT study	Patients followed for 12 months	Standardised assessment tools	Points
Krogh et al., (2023), Denmark	-	-	-	+	-	+	+	2
Petrover et al., (2017), France	+	-	+	+	-	-	-	3
Rojo-Manaute, et al., (2016), Spain	-	+	-	+	+	+	+	5
Nakamichi et al., (2010), Japan	-	-	-	-	-	-	+	1
McShane et al., (2012), USA	-	-	-	+	-	+	+	3
Guo et al., (2014), USA	-	-	-	-	-	-	+	1
McCormack et al., (2012), USA	-	-	+	-	-	-	-	1
Guo et al., (2017), USA	+	-	-	-	-	+	+	3
Cano et al., (2024), USA	+	-	-	+	-	+	+	4
Capa-Grasa et al., (2014), Spain	-	-	-	+	-	-	+	2
Henning et al., (2018), USA	-	-	-	-	-	-	+	1
Burnham et al., (2021), Canada	-	-	-	+	-	-	+	2
Wang et al., (2014), Taiwan	-	-	-	-		+	+	3
Eberlina et al., (2023), USA	+	+	-	-	+	-	+	4

Kim et al., (2024), Republic of Korea	+	-	-	-	-	+	+	3
Bergum et al., (2022), USA	-	-	-	-	-	+	+	2
Li et al ., (2019), China	-	+	-	+	-	+	-	3
Joseph et al., (2020), USA	-	-	-	-	-	-	+	1
Akhoondinasab et al., (2024), Iran	-	+	+	-	+	-	+	4
Chen et al., (2021), China	-	-	-	+	+	+	-	3
Pistorio et al., (2024), USA	+	-	-	-	-	-	+	2
Nicholas et al., (2024), USA	-	-	-	-	-	-	+	1
Kamel et al., (2020), USA	-	-	-	-	-	+	+	2
Leiby et al., (2022), USA	-	-	-	-	-	+	+	2
Chappell et al., (2020), USA	-	-	-	-	-	-	+	1
Hebbard et al., (2021), Australia	+	-	-	-	-	-	+	2
Chern et al., (2015), Taiwan	-	-	-	+	-	-	+	2
Luanchumroen et al., (2019), Thailand	-	-	+	+	-	-	+	3
