# Supplementary material for: Safety and Effectiveness of Ultrasound-Guided Carpal Tunnel Release: A Systematic Review
Source: J Clin Med. 2026 Aug 28;15(17):6675. doi: 10.3390/jcm15176675 (PMC13566160; doi:10.3390/jcm15176675)
Supplement: Supplementary file 1 [file jcm-15-06675-s001.zip › Table S2. Complications by Technique.pdf]

Supplementary Table S2. Complication rate by ultrasound-guided carpal tunnel release technique

Reference numbers correspond to the manuscript reference list. Bracketed superscripts indicate the study/studies contributing to each row; where a study reported two treatment arms, hand counts are given for the relevant arm only. Overall across all CTR-US-family techniques (UMIU-CTR + TCTR + PCTR + device-assisted CTR-US + MICTR): ~1,928 hands, ~12–13 with a reported complication (≈0.6–0.7%), no permanent nerve or vascular injuries, and one reoperation (MICTR/MANOS cohort).

Technique	Studies (n)	Total hands	Hands with ≥ 1 complication	Complication rate	Nature of complications
**UMIU-CTR**	3 [12,13,14]	~76	0	**0%**	None reported
**TCTR**	5 [21,22,23,29,32]	~391	2–3	**~0.5–0.8%**	Transient wrist/hand swelling, restricted finger flexion, pillar pain — all self-resolving within days to weeks
**PCTR**	5 [18,19,20,27,39]	313	5	**1.6%**	Entirely driven by one study (Luanchumroen [39]: 5/19 hands, 26.3%, transient paresthesia); the other four PCTR studies (294 hands) reported zero complications
**CTR-US (device-assisted: MicroKnife / SX-One / MICROi-Blade)**	11 [15,24,25,30,31,33,34,35,36,37,38]	1,092	≥5 (+unquantified)	**≥0.5%**	Transient nerve inflammation, nerve contusion (resolved), MRSA wound infection, CRPS; Hebbard [38] reports transient numbness/swelling in an unspecified number of the 166 hands
**MICTR (MANOS device)**	1 [22]	56	5	**8.9%**	Highest rate in the review: injection-site redness/swelling (2), persistent swelling (1), persistent numbness (1), 1 reoperation for incomplete TCL release
Comparator: OCTR / mini-OCTR / MOCTR	4 [7,13,20,28]	~213	4	**~1.9%**	CRPS, superficial infection, wound dehiscence, symptom recurrence requiring steroid injection
Comparator: ECTR / MECTR	2 [7,10]	~138	0	**0%**	None reported
