# Supplementary material for: Safety and Effectiveness of Ultrasound-Guided Carpal Tunnel Release: A Systematic Review
Source: J Clin Med. 2026 Aug 28;15(17):6675. doi: 10.3390/jcm15176675 (PMC13566160; doi:10.3390/jcm15176675)
Supplement: Supplementary file 1 [file jcm-15-06675-s001.zip › Table S3. CTR-US Timeline.pdf]

**Table S3.** Timeline of the evolution of ultrasound-guided carpal tunnel release techniques and devices.

| Period | Technique / device (manufacturer) | Access port | Principal technical advance | Studies included in this review |
|---|---|---|---|---|
| 2010 | Blade-and-guide PCTR (custom no. 138 blade with guide and holder) | 4 mm | First ultrasound-guided percutaneous release evaluated against a mini-open control; established feasibility of instrument-guided transection. | Nakamichi et al., 2010 [28] |
| 2012 | Needle-based PCTR (18-G needle, no dedicated device) | 0.5–1.2 mm | Release using only off-the-shelf instruments; demonstrated that no proprietary hardware is required. | McShane et al., 2012 [29] |
| 2012 | MICTR with the MANOS device (Thayer Intellectual Property) | 2.1 mm | First purpose-built carpal tunnel release device combining ultrasound visibility with intraoperative nerve stimulation. No longer commercially available. | McCormack et al., 2012 [30] |
| 2014–2016 | Ultra-minimally invasive CTR (UMIU-CTR / Ultra-MIS; 3.0 mm Acufex hook knife, Smith & Nephew) | ≤ 1 mm | Intratunnel positioning of the hook knife with the release restricted to the deepest fibrous layer, sparing the intermediate layer and palmar innervation. | Capa-Grasa et al., 2014 [13]; Rojo-Manaute et al., 2016 [14]; Krogh et al., 2023 [15] |
| 2014–2015 | Thread carpal tunnel release (TCTR; looped cutting thread, no blade) | 0.4–1.2 mm | Blade-free transection performed entirely through needle punctures, enabling office-based release without a skin incision. | Guo et al., 2014/2015 [18]; Guo et al., 2017 [17]; Burnham et al., 2021 [33]; Kim et al., 2024 [36]; Akhoondinasab et al., 2024 [39] |
| 2015–2019 | Hook-knife PCTR variants (custom hook knife ± dilating probe) | 0.8–5 mm | Formalisation of the transverse and longitudinal “safe zone” concept; extension of the technique to larger series and to special populations (haemodialysis). | Chern et al., 2015 [16]; Petrover et al., 2017 [27]; Wang et al. [34]; Luanchumroen et al., 2019 [43] |
| 2018 | SX-One MicroKnife / UltraGuideCTR (Sonex Health) | 4–6 mm | Retractable retrograde cutting blade housed between inflatable balloons that displace the median nerve and vessels away from the blade; the most extensively reported device to date. | Henning et al., 2018 [32]; Joseph et al., 2020 [38]; Kamel et al., 2020 [21]; Chappell et al., 2020 [41]; Bergum et al., 2022 [37]; Leiby et al., 2022 [40]; Eberlin et al., 2023 [35]; Nicholas et al. [19]; Cano et al., 2024 [31]; Pistorio et al., 2024 [20] |
| 2018–2021 | MICROi-Blade (Summit Medical Products) | Needle-mounted blade | Retractable needle-mounted blade cutting retrograde with digital counter-pressure; a general-purpose cutting tool also applicable to other releases. Clinical use from June 2018; case series published 2021. | Hebbard et al., 2021 [42] |
| 2023–2024 | Consolidation of evidence rather than new hardware | — | First multicentre randomised comparison of CTR-US with mini-open release, and the first cohorts reporting follow-up beyond two years. | Eberlin et al., 2023 [35]; Cano et al., 2024 [31]; Kim et al., 2024 [36] |

**Abbreviations:** CTR-US — carpal tunnel release with ultrasound guidance; MICTR — minimally invasive carpal tunnel release; PCTR — percutaneous carpal tunnel release; TCTR — thread carpal tunnel release; UMIU-CTR — ultra-minimally invasive ultrasound-guided carpal tunnel release; Ultra-MIS — ultra-minimally invasive surgery.
